# The establishment of species-specific primers for the molecular identification of ten stored-product psocids based on ITS2 rDNA

**DOI:** 10.1038/srep21022

**Published:** 2016-02-16

**Authors:** Zi-Hua Zhao, Bing-Yi Cui, Zhi-Hong Li, Fan Jiang, Qian-Qian Yang, Zuzana Kučerová, Václav Stejskal, George Opit, Yang Cao, Fu-Jun Li

**Affiliations:** 1 Department of Entomology, College of Plant Protection, China Agricultural University, Beijing 100193, China; 2 Crop Research Institute, Drnovská 507, 161 06 Prague 6, Czech Republic; 3 Department of Entomology and Plant Pathology, 127 Noble Research Center, Oklahoma State University, Stillwater, OK 74078, USA; 4 Academy of State Administration of Grain, Beijing 100037, China; 5 Institute of Plant Quarantine Research, Chinese Academy of Inspection and Quarantine, Beijing 100176, China; 6 College of Life Science, China Jiliang University, Hangzhou 310018, China

## Abstract

Psocids are important stored product pests found worldwide that can be spread through grain trade. Most stored-product psocids, including eggs, nymphs, and adults, are very small (~1 mm) and difficult to identify morphologically. Here, we collected 10 economically important stored-product *Liposcelis* spp. psocids (*L. bostrychophila*, *L. entomophila*, *L. decolor*, *L. paeta*, *L. brunnea*, *L. corrodens*, *L. mendax*, *L. rufa*, *L. pearmani*, and *L. tricolor*) from 35 geographical locations in 5 countries (China, Czech Republic, Denmark, Germany, and the United States). The ITS2 rDNA gene was extracted and sequenced. The interspecific genetic distance of the stored-product psocids was significantly higher than the intraspecific genetic distance according to the barcoding gap analysis. Ten pairs of species-specific primers based on the ITS2 rDNA were developed for psocid identification. The sensitivity estimation indicated that the species-specific primers could correctly amplify the target ITS2 gene and successfully identify psocids at 1.0 ng/mL. Additionally, these species-specific primers could quantify specificity and identify 10 stored-product psocids; this approach could also be used to accurately identify other stored-product psocids. This work provides a practical approach for the precise examination of 10 stored-product psocid species and also contributes to the development of an identification method using ITS2 rDNA.

Stored-product psocids (Psocoptera: Liposcelididae) are known by the common names booklice, barklice, or dustlice and belong to the genus *Liposcelis*. Psocids are small in body size (~1 mm) and have a wide geographical distribution^1^. Stored-product psocids are closely associated with cereal grains and their processed products; their worldwide economic importance as pests stems from this association^2^. In recent years, the economic importance of stored-product psocids has rapidly increased^3^. Typically, stored-product psocid species infestations involve multiple species that multiply rapidly and disperse easily^4^. Currently, there are more than 120 *Liposcelis* species (booklice) in the world. Among them, 10 species, *Liposcelis bostrychophila* (Badonnel), *L. entomophila* (Enderlein), *L. decolor* (Pearman), *L. paeta* (Pearman), *L. brunnea* (Motschulshy), *L. corrodens* (Motschulsky), *L. mendax* (Pearman), *L. rufa* (Broadhead), *L. pearmani* (Lienhard), and *L. tricolor* (Badonnel), are economically important stored pests that can also be spread through grain trade^5^,^6^. In China, stored-product psocids are often intercepted at entry ports^7^. According to the literature, Stored-product psocids are often intercepted in seaports in China (approximately 3000 times/year). However, identification of all stages of stored-product psocids using morphological characteristics is very difficult due to their small body size^3^. Therefore, accurate identification methods are urgently needed to guarantee the safety and reliability of the grain trade and to facilitate effective plant quarantine^8^.

Many types of molecular techniques have been used to identify insect species, including restriction fragment length polymorphism (RFLP), amplified fragment length polymorphism (AFLP), random amplified polymorphic DNA (RAPD), single-strand conformation polymorphism (SSCP), DNA sequence analysis, and species-specific primer PCR (SS-PCR) methods^9^,^10^,^11^,^12^. Among these techniques, species-specific primer PCR is a popular technology for species identification due to its high accuracy, sensitivity and convenience^13^. Both mtDNA (mitochondrial DNA) and rDNA (ribosomal DNA) are species-specific and can be used as molecular markers for species identification^14^,^15^. In insects, rDNA is composed of a coding region (18 S rRNA, 5.8 S rRNA, and 28 S rRNA), an internal transcribed spacer (ITS), and a non-transcribed spacer (NTS)^16^. Internal transcribed spacer (ITSs) are moderately conserved and are suitable for species identification. Non-transcribed spacer (NTS) are highly variable and are suitable for the examination of macroevolution^17^.

There are two ITS’s (ITS1 and ITS2) in eukaryotes, which are two spatially distinct spacers^18^. ITS1 is located between 18 S rDNA and 5.8 S rDNA, whereas ITS2 is located between 5.8 S rDNA and 28 S rDNA^19^. ITS is a highly repetitive sequence that is removed after DNA transcription^20^. Additionally, 5.8 S rDNA, 18 S rDNA, and 28 S rDNA are gene conservative region and can be used to design both 3’ and 5’ primers. Generally, ITS2 is conserved within a species but significantly varies among species^21^. In recent years, ITS2 has been identified as an important gene in taxonomy, phylogenetic development, and species evolution^22^. Young *et al.* (2004) reported that ITS2 can be used as molecular markers in the fruit fly and can also be used to construct a phylogenetic tree^19^,^23^.

Although the rapid identification of stored-product psocids has received substantial attention in recent years, systematic molecular identification of stored-product psocids is lacking^24^. Currently, there are some molecular markers (e.g., COI, 16 S rDNA, and microsatellite) and technology (e.g., RFLP, DNA barcoding, and species-specific primers) for the identification of stored-product psocid species^4^. However, molecular identification of only a few psocid species has been conducted^5^,^7^. In our previous studies, we developed a species-specific molecular marker to identify *L. entomophila*, *L. reticulatus*, *L. corrodens*, *L. bostrychophila*, *L. decolor*, and *L. paeta* based on 16 S rDNA, mtDNA, CO1 gene, and PCR-RFLP analysis^25^,^26^. Wei *et al.* found that the ITS gene could be used to study gene structure and phylogenesis of stored-product psocid species. Additionally, the differences of ITS sequence and species-specific primers were also found among 6 stored-product psocid species (*L. bostrychophila*, *L. entomophila*, *L. decolor*, *L. paeta*, *L. tricolor*, and *L. yunnaniensis*) based on multiple PCR techniques^7^. Until now, there were no reports to design the species-specific primers of ITS2 for *L. brunnea*, *L. corrodens*, *L. mendax*, *L. rufa*, and *L. pearmani*. In this study, we sequenced the ITS2 rDNA of 10 psocid species, tested the effectiveness of ITS2 rDNA for psocid identification using a barcode gap analysis and established a barcode library. Then, species-specific primers based on ITS2 were developed to conduct stored-product psocid identification. These primers were also applied in practice to identify psocids intercepted in ports in China. Our results suggest that our molecular identification approach for efficient identification on psocid species can provide technological support for plant quarantine and stored commodity protection purposes. This work is also an essential prerequisite enabling determination of the geographic origin of *Liposcelis* infestations.

## Results

### ITS2 sequencing

**Figure 1:**
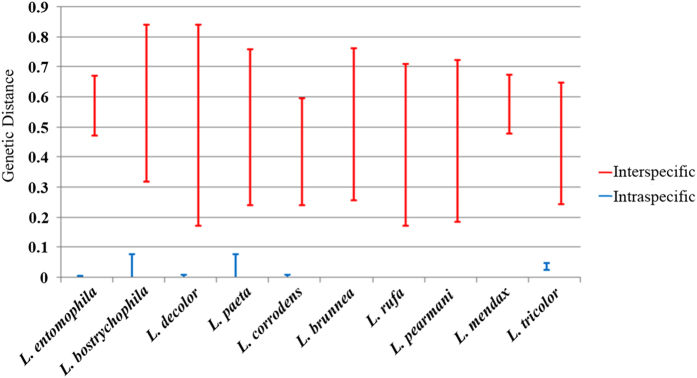
The intraspecies and intraspecific genetic distance of ITS2 gene in 10 stored-product psocids.

The concentration of total DNA for a stored-product psocid individual was 60-80 ng/uL after extraction, which was suitable for further PCR amplification. Additionally, the ITS2 gene of all 10 stored-product psocid species could be amplified successfully and was then examined using agarose gel electrophoresis (Supplemental Material Appendix Table S1). The lengths of the ITS2 gene ranged from 293 bp to 614 bp depending on the species (Supplemental Material Appendix Table S2). After sequencing, more than 180 ITS2 sequences of the 10 stored-product psocid species were obtained, of which 53 sequences were specific to ITS2. The ITS2 lengths of the different species varied greatly. *L. brunnea* had the longest ITS2 sequence (614 bp), whereas *L. bostrychophila* had the shortest ITS2 sequence (293 bp) (Supplemental Material Appendix Table S2). Furthermore, the interspecies genetic distance was relatively large compared with the intraspecies genetic distance. *L. bostrychophila* had the largest intraspecific genetic distance (0.05), whereas *L. rufa* had the lowest intraspecific genetic distance (0.00) (Fig. 1, *t*_10.22_ = −22.373, *p* < 0.001). Therefore, the minimum interspecific genetic distance of each stored-product psocid species was significantly higher than the maximum intraspecific genetic distance, which indicated that ITS2 sequence can be used for molecular identification by using barcoding gaps at the species level (Fig. 1). Additionally, an ITS2 rDNA sequence information library for the 10 stored-product psocid species was established based on these 53 specific sequences (Supplemental Material Appendix Table S3).

### Specificity and sensitivity Species-specific primers

Ten species-specific primers were successfully developed based on ITS2 rDNA using Primer Premier 5.0 (Table 1). All 10 species-specific primers could specifically amplify the ITS2 gene sequence of *L. bostrychophila*, *L. entomophila*, *L. decolor*, *L. paeta*, *L. brunnea*, *L. corrodens*, *L. mendax*, *L. rufa*, *L. pearmani*, and *L. tricolor* (Supplemental Material Appendix Figures S1-S10).

PCR with the 10 species-specific primers in a series of seven concentrations for a single stored-product psocid was conducted to test the sensitivity of the primers. The DNA template concentrations of the stored-product psocid were 0.001, 0.01, 0.1, 1, 10, 20, and 40 ng/mL. The results showed that all ten species-specific primers could amplify the positive electrophoresis band at 1 ng/mL (Supplemental Material Appendix Figures S11-S20). Additionally, all ten species-specific primers indicated that the electrophoresis bands in the agarose gel appeared clearer with increasing concentrations of the DNA template (Supplemental Material Appendix Figures S11-S20).

### Practice of species-specific primers

**Figure 2:**
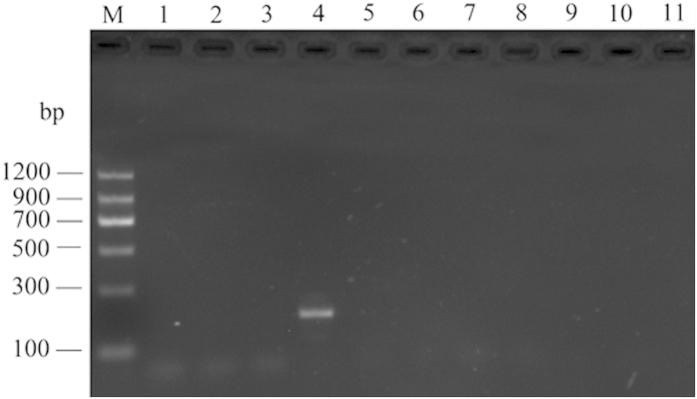
The application of species-specific primers for rapid species identification with of electrophoresed ITS2 rDNA PCR amplification products from 10 stored-product psocids (M: DNA Marker II; 1: *L. entomophila*; 2: *L. bostrychophila*; 3: *L. paeta*; 4: *L. decolor*; 5: *L. corrodens*; 6: *L. brunnea*; 7: *L. rufa*; 8: *L. pearmani*; 9: *L. mendax*; 10: *L. tricolor*; 11: Negative control).

To examine the specificity of these 10 species-specific primers in practice, the stored-product psocid from a grain depot in Yantai of the Shandong province was amplified by *L. decolor*. A single electrophoresis band was obtained in the electrophoresis of 164LDeF and 319LDeR (*L. decolor*), and no bands were evident for any of the other species tested (Fig. 2). The similarity between this sequence and the corresponding sequence of *L. decolor* was 98.98%. Additionally, the result of the morphological identification also indicated that stored-product psocid in Yantai of the Shandong province is *L. decolor*, which is coincident to the results of molecular identification.

## Discussion

In our study, 10 pairs of species-specific primers based on the ITS2 rDNA could be used in identify 10 economically important stored-product psocid species from 35 geographical populations, which indicated that ITS2 rDNA was also an effective molecular marker for identifying stored-product psocid. The total nucleotide sequences of the ITS1-5.8 S-ITS2 region in six *Liposcelis* species from 16 locations of China have been determined and the suitability of ITS1-5.8 S-ITS2 region for phylogenetic inference analysis in the *Liposcelis* species has been estimated. Additionally, Wei *et al.* (2011) provided six pair of primers for amplifying ITS2 region of six species, which were different to the species-specific primers in our experiment^7^. To the best of our knowledge, this is the first study where 10 stored-product psocids have been identified using ITS2 rDNA. We also provided the total ITS2 sequences, which will contribute important data for additional phylogenetic studies of stored-product psocids. Furthermore, the efficiency and specificity of our 10 pairs of species-specific primers still need to further evaluate on other stored-product psocid species or geographical populations in future research.

Recently, stored-product psocids have been spread all over the world and cause great damage on stored products^7^,^27^. Molecular identification of stored-product psocids is crucial for plant quarantine and stored commodity protection purposes especially when only juvenile insects (nymphs) are available for identification^28^. Accurate molecular identification of stored-product psocids facilitates trade^29^. Over the past few decades, molecular technology has been increasingly been used for species identification, especially the CO I gene in the chondriosome. Additionally, DNA barcoding has been standardized for easy species-specific amplification of gene fragments^30^,^31^. Several studies have reported that the CO I gene in the chondriosome can be used to identify species, and this finding has been widely applied to DNA barcoding^32^,^33^. However, DNA barcoding based on CO I also has some limitation, especially for geographical populations of the same species^34^. In our experiment, the IST2 rDNA gene was selected for the design species-specific primers. At the sub-species level, the IST2 rDNA gene may be an important molecular marker that can also be used to distinguish geographical populations of insects.

The intraspecific barcoding gap of the IST2 rDNA that we identified within each species of the stored-product psocids was not significant enough to conduct population genetic analysis because many ITS2 sequences of one species from multiple geographical populations were the same due to the intraspecific conservation. However, the interspecific barcoding gap among the stored-product psocids was 50-fold higher than the intraspecific barcoding gap of the stored-product psocids. These results indicated that the ITS2 gene could be used for species identification, as well as phylogenetic delineation and association studies of other stored-product psocids^35^. Very few ITS2 gene sequences of stored-product psocids are present in the literature, and our submission of 53 ITS2 sequences will significantly enlarge this database with the addition of an accessible accurate identification system^34^.

No non-target PCR products could be amplified from non-target stored-product psocid species, which indicated that the species-specific primers were highly accurate at identifying stored-product psocids in this experiment^13^,^15^. Here, 10 species-specific primers of ITS2 rDNA were developed based on 35 geographical populations in China, Czech, Denmark, Germany, and the United States. We also submitted 53 sequences of the ITS2 rDNA to GenBank and obtained 53 accession numbers. We examined the sensitivity of these 10 species-specific primers, the results of which indicate that these primers show good specificity and sensitivity. In summary, reliable molecular markers of stored-product psocids are urgently needed to accurately identify species, particularly when morphological approaches cannot be used, for example, for the identification of nymphs, males, super species, and specific geographical strains^28^. Multiple molecular markers would ultimately lead to a more accurate molecular diagnostic approach with a wide range of applications in practice^35^,^36^, including species identification, quarantine confirmation, international trade regulation, and pest control, i.e., assisting in the determination of the origins of infestations^25^.

## Materials and Methods

### Study species

We collected 10 stored-product psocid species, including 35 geographical populations from 5 countries (China, Czech Republic, Denmark, Germany, and the United States). All 35 geographical populations were first identified by a taxonomist (Dr. Charles Lienhard) using morphological characteristics. Then, all of the stored-product psocids were cultured in a Plant Quarantine and Invasion Biology lab at China Agricultural University (Table 2). An artificial diet (wheat flour, yeast powder, and dried skimmed milk at 10:1:1 wt/wt) was used to feed these 10 stored-product psocid species. The temperature and humidity were maintained at 28.5 °C and 70% RH, respectively.

### DNA extraction

An entire adult stored-product psocid was used for total DNA extraction with a Tiangen DNA Extraction Kit. The extraction process was as follows: 1) select an adult psocid, rinse with absolute ethyl alcohol, and dry on filter paper; 2) put the dry psocid into a 1.5 mL Eppendorf (EP) tube, add 10 μL of buffer GA, and grind with a grinding rod; 3) add 170 μL of GA into the 1.5 mL EP tube containing the psocid solution and centrifuge for approximately 15 seconds (12000 rpm/s); 4) add 10 μL of protease K into the EP tube, place in a water bath at 56 °C for 1 hour and centrifuge for approximately 15 seconds (12000 rpm/s); 5) add 200 μL of GB and 1 μL of carrier RNA into the EP tube, place in a water bath at 70 °C for 10 minutes and centrifuge for approximately 30 seconds (12000 rpm/s); 6) add 200 μL of absolute ethyl alcohol, shake for 15 seconds, centrifuge for 60 seconds, set the CR2 aside for 5 minutes, and then transfer to an adsorbing column (CR2) and centrifuge for approximately 30 seconds (12000 rpm/s); 7) add 500 μL of GD into the CR2 and centrifuge for approximately 30 seconds (12000 rpm/s) before discarding the solution; 8) add 700 μL of poaching liquid (PW) into the CR2 and centrifuge for approximately 30 seconds (12000 rpm/s) and then discard the solution; 9) add 500 μL of poaching liquid (PW) to CR2, centrifuge for approximately 30 seconds (12000 rpm/s) and then discard the solution; 10) centrifuge the CR2 for approximately 2 minutes (12000 rpm/s), open the lid and set the CR2 aside for 5 minutes; 11) put the CR2 into another clean 1.5 mL Eppendorf (EP) tube, add 30 μL of hyperpure water into the EP tube, set the CR2 aside for 5 minutes and centrifuge for approximately 2 minutes (12000 rpm/s); 13) store the DNA in the EP tube at −20 °C. Additionally, 6 individuals for each geographical populations of 10 stored-product psocids were used to conduct DNA extraction.

### PCR amplification

One pair of primers was selected for amplifying the ITS2 gene of 10 stored-product psocids based on a screening process (Supplemental Material Appendix Table S1). Oligo synthesis was conducted by the Beijing Aoke Biotechnology Company. A 50 μL PCR reaction system was used, which contained 3 μL of DNA template, 25 μL of Premix EX Taq^TM^, 20 μL of ddH_2_O, 1 μL of forward primer, and 1 μL of reverse primer. The PCR reaction conditions included a 4 min DNA pre-denaturation at 94 °C and 30 amplification cycles (30 s for denaturation at 94 °C, 30 s for DNA annealing at 52 °C, and 30 s for extension at 72 °C). Finally, agarose gel electrophoresis was used to examine the reaction product (stained with ethidium bromide) on a GEL imaging system (Gel Logical Pro 212).

### Clone and sequencing

All PCR products were subjected to iontophoretic separation in a 2.0% agarose gel. Gel purification was performed to obtain the target fragments. pMD18-T was used for DNA ligation in a 10 μL reaction (5 μL of buffer solution, 1 μL of pMD18-T vector, 2 μL of target fragments, 0.5 μL of T4 DNA ligase, and 1.5 μL of ddH_2_O). Then, the ligated vector was used to transform DH5ą of *Escherichia coli*, which was cultured on the LB medium. Subsequently, positive clones were selected for PCR amplification and bidirectional sequencing (3730XL DNA Genetic Analyser, ABI). The amplification products (ITS2) of each psocid species were kept in the refrigerator at −20 °C. DNAMAN (Lynnon BioSoft, Version 5.2, 2001) was used for a bidirectional combine and compare to correct the sequences. The gene sequences of 10 stored-product psocid species were submitted to GenBank.

### Barcode gap analysis and species-specific primers design

After sequencing, the intra- and inter-specific genetic distances of the ITS2 gene for the 10 stored-product psocid species were analyzed using MEGA 5.0. The minimum, maximum and mean genetic distances of the intra-specific and inter-specific variations of stored-product psocids were estimated using Excel. Finally, a barcoding gap analysis was performed to evaluate the utility of ITS2 gene for 10 stored-product psocids identification. Moreover, t-test (*Welch Two Sample t-test*) was used to examine the significance of difference between intra- and inter-specific genetic distances.

The alignment of the ITS2 genes of the 10 stored-product psocids was performed using MEGA 5.0 software, and single nucleotide polymorphism (SNP) sites were identified manually based on the alignment result. Species-specific primers for each stored-product psocid species were designed against the SNP region using Primer Premier 5.0. Oligo 7 was used to estimate all indices (primer mismatch, dipolymer, and hairpin structure) of the species-specific primers. In addition, the primers were designed based on the following standard criteria: the 3’ Delta G absolute value of the forward and reverse primers should not exceed 9; the number of combined base pairs, which may form dipolymer or hairpin structures, should not exceed 3; the percentages of G and C should range from 30 to 70%; and error initiation efficiency should not exceed 100. Finally, Oligo 7 provided the melting, annealing, and extending temperature for each primer. We designed several species-specific primers for each stored-product psocid species. Only one pair of optimal primers for each species was reserved for stored-product psocid identification after selection by a specificity test. The oligos were synthesized by Beijing Aoke Biotechnology Company.

### Specificity test of the species-specific primers

The specificity of the 10 stored-product psocid species-specific primers was tested by performing PCR assays on ten species from 35 geographical populations. PCR amplification using the species-specific primers in a total reaction volume of 50 μL was performed using the following components: Premix EX Taq^TM^ 25 μL, ddH_2_OμL, DNA Template 2 μL, upstream primer 1 μL, downstream primer 1 μL. The PCR cycling conditions were 4 min DNA pre-denaturation at 94 °C and 30 amplification cycles (30 s for denaturation at 94 °C, 30 s for DNA annealing at 53 °C, and 30 s for extension at 72 °C). The PCR products (5 μl) from each individual were mixed with 1 μl of loading buffer, analyzed by gel electrophoresis on a 2% agarose gel in 1 × TAE buffer, and then visualized under UV light after EB staining. To confirm the amplified products generated by the 10 stored-product psocid species-specific primers, each positive band product was sequenced by Beijing Aoke Biotechnology Co. Ltd, and the results were then compared with known sequences by searching the NCBI and BOLD databases. Furthermore, 3 individuals of per population and populations per species were used to examine the specificity of species-specific primers.

### Sensitivity estimation of the species-specific primers

Sensitivity tests for these ten species-specific primers were carried out in a series of seven concentrations ranging from 0.001 ng/mL to 40 ng/mL and a negative control. The same reaction mixture and cycling processes described above for DNA amplification were used for the sensitivity tests.

### The application of species-specific primers for psocid identification

These 10 species-specific primers were also applied in to identify stored-product psocid species from a grain depot in Yantai of the Shandong province. Both morphological characteristics and PCR using species-specific primers were used to identify the intercepted stored-product psocid.

## Additional Information

**How to cite this article**: Zhao, Z.-H. *et al.* The establishment of species-specific primers for the molecular identification of ten stored-product psocids based on ITS2 rDNA. *Sci. Rep.* **6**, 21022; doi: 10.1038/srep21022 (2016).

## Supplementary Material

Supplementary Information

## Figures and Tables

**Table 1:** The species-specific primers for ITS2 rDNA amplification of 10 stored-product psocids.

Species	Primer	5′-3′	Length	Tm(°C)	Target fragment
*L. entomophila*	21LEnF	ACATTGCTGGGTTAGGATTCA	21	56.	188
	208LEnR-2	CTTCCGAACTCTGTAATTTGT	21	54.1	
*L. bostrychophila*	LBF	CGCACATTGCCGAGCTAGGAT	21	62.3	177
	LBR	CGCTCCTTGTATTCTCGTTCT	21	58.3	
*L. decolor*	164LDeF	GAAATGCACTAGAACCGAGA	20	53.5	175
	319LDeR	ATATATTGAACGTGACGAGT	20	52	
*L. paeta*	LPa15F	GAACGCACATTGCCGAGTTTT	21	59.7	186
	LPa180R	ACCGTCCGATGCAGTTACCCTA	22	65.1	
*L. corrodens*	LC170F	CGAGGAAATCTCACAAGACG	20	55.8	128
	LC277R	GTTCTCTACAATCCGTTTGGT	21	55.1	
*L. brunnea*	LBr350F	ACCGAGATCCTTGTTACGAA	20	57.6	248
	LBr577R	GCCGGAATTCTGTTTTACGAG	21	56.4	
*L. rufa*	78LRuF-3	AATGTTAAATGTCGGAACGCT	21	54.1	199
	276LRuR-3	TTACGACCTATCGGTACGCTA	21	58	
*L. pearmani*	186LPeF	ATGCACGAAACGAGAATTCTCA	22	56.3	251
	436LPeR	AACGGCTACCATTCTTCAAAC	21	56.3	
*L. mendax*	LM60F	GTCTGAGGGTCGGTGTTTGCT	21	61.9	185
	LM224R	AAGTCCGTCAACGCTGCTCTT	21	62.2	
*L. tricolor*	LTri20F	CACATTGCCGAACTTTGAATT	21	56.4	250
	LTri249R	TCTACTATCCGTTTGGTTTAC	21	52.6	

**Table 2:** The 35 geographical populations of 10 stored-product psocids from 5 countries

Species	Geographical population	Locations
*Liposcelis entomophila*	*L. entomophila*_BJ-China	Beijing, China
	*L. entomophila*_HuB-China	Hubei province, China
	*L. entomophila*_GX-China	Guangxi province, China
	*L. entomophila*_SD-China	Shandong province, China
*Liposcelis bostrychophila*	*L. bostrychophila*_BJ-China	Beijing, P. R. China
	*L. bostrychophila*_GX-China	Guangxi province. China
	*L. bostrychophila*_GZ-China	Guangdong province, China
	*L. bostrychophila*_HeN-China	Henan province, China
	*L. bostrychophila*_CQ-China	Chongqing, China
	*L. bostrychophila*_P-CZ	Prague, Czech Republic
	*L. bostrychophila*_CZ	Bohemia, Czech Republic
	*L. bostrychophila*_USA	Manhattan,USA
	*L. bostrychophila*_GER	Berlin, Germany
*Liposcelis decolor*	*L. decolor*_CQ- China	Chongqing, China
	*L. decolor*_YN-China	Yunnan province, China
	*L. decolor*_P-CZ	Prague, Czech Republic
	*L. decolor*_USA	USA
*Liposcelis paeta*_	*L. paeta*_USA	USA
	*L. paeta*_HeB-China	Hebei province, China
	*L. paeta*_SDT-China	Shandong province, China
	*L. paeta*_SDC-China	Shandong province, China
	*L. paeta*_ZJ-China	Zhejiang province, China
	*L. paeta*_HuB-China	Hubei province, China
	*L. paeta*_HeN- China	Henan province, China
	*L. paeta*_CZ	Prague, Czech Republic
*Liposcelis corrodens*	*L. corrodens*_CZ	Prague, Czech Republic
	*L. corrodens*_DMK	Danmark
	*L. corrodens*_ USA	USA
	*L. corrodens*_USA	USA
*Liposcelis brunnea*	*L. brunnea*_P-CZ	Prague, Czech Republic
	*L. brunnea*_USA	USA
*Liposcelis mendax*	*L. mendax*_JS-China	Jiangsu province, China
*Liposcelis pearmani*	*L. pearmani*_USA	USA
*Liposcelis tricolor*	*L. tricolor*_SD-China	Shandong provience, China
*Liposcelis rufa*	*L. rufa*_USA	USA
