# Supplementary material for: The establishment of species-specific primers for the molecular identification of ten stored-product psocids based on ITS2 rDNA
Source: Sci Rep. 2016 Feb 16;6:21022. doi: 10.1038/srep21022 (PMC4754681; doi:10.1038/srep21022)
Supplement: Supplementary Information [file srep21022-s1.pdf]

The establishment of species-specific primers for the molecular identification of ten stored-product psocids based on ITS2 rDNA

Zi-Hua Zhao[1,§], Bing-Yi Cui[1,§], Zhi-Hong Li[1,*], Fan Jiang[1,5], Qian-Qian Yang[1,6], Zuzana Kučerová[2], Václav Stejskal[2], George Opit[3], Yang Cao[4], Fu-Jun Li[4]

[1] Department of Entomology, College of Plant Protection, China Agricultural University, Beijing 100193, China;

[2] Crop Research Institute, Drnovská 507, 161 06 Prague 6, Czech Republic;

[3] Department of Entomology and Plant Pathology, 127 Noble Research Center, Oklahoma State University, Stillwater, OK 74078, USA;

[4] Academy of State Administration of Grain, Beijing 100037, China;

[5] Institute of Plant Quarantine Research, Chinese Academy of Inspection and Quarantine, Beijing 100176, China;

[6] College of Life Science, China Jiliang University, Hangzhou 310018, China;

[§] These two authors (Zi-Hua Zhao and Bing-Yi Cui) contributed equally to this research.
Address: No. 2 Yuanmingyuan West Road, Haidian District, Beijing 100193, China
[*] Corresponding author: lizh@cau.edu.cn (Zhi-Hong Li)

Supplement Material Appendix

Table S1 The primers for the ITS2 gene amplification of 10 stored-product psocids

Table S2 The ITS2 gene sequence of 10 stored-product psocids (The sequences highlighted in red indicate species-specific primers for ten stored-product psocids)

Table S3 The number of 53 specific sequences submitted to Genebank

Figure S1 The electrophoresis strip for specificity estimation of species-specific primers (LBF and LBR) of *L. bostrychophila* (M: D2000 DNA Marker; 1: *L. bostrychophila* (Guangxi); 2: *L. bostrychophila* (Henan); 3: *L. bostrychophila* (Chongqing); 4: *L. bostrychophila* (Berlin); 5: *L. bostrychophila* (USA); 6: *L. bostrychophila* (Prague); 7 *L. bostrychophila* (Beijing); 8: *L. bostrychophila* (Guangzhou); 9: *L. entomophila* (Shandong); 10: *L. decolor* (Yunnan); 11: *L. paeta* (Zhejiang); 12: *L. corrodens* (USA); 13: *L. brunnea* (Prague); 14: *L. rufa* (USA); 15: *L. pearmani* (USA); 16: *L. mendax* (Jiangsu); 17: *L. tricolor* (Shandong); CK: Negative control)

Figure S2 The electrophoresis strip for specificity estimation of species-specific primers (21LEnF and 208LEnR-2) of *L. Entomophila* (M: DNA Marker Ⅱ; 1: *L. entomophila* (Beijing); 2: *L. entomophila* (Guangxi); 3: *L. entomophila* (Prague); 4: *L. entomophila* (Chongqing); 5: *L. bostrychophila* (Guangxi); 6: *L. bostrychophila* (Yunnan); 7: *L. Decolor* (Yunnan); 8: *L. corrodens* (USA); 9: *L. brunnea* (Prague); 10: *L. rufa* (USA); 11: *L. pearmani* (USA); 12: *L. mendax* (Jiangsu); 13: *L. tricolor*(Shandong); CK: Negative control)

Figure S3 The electrophoresis strip for specificity estimation of species-specific

primers (164LDeF and 319LDeR) of *L. decolor* (M: DNA Marker Ⅱ; 1 and 2: *L. Decolor* (Yunnan); 3 and 4: *L. Decolor* (Chongqing); 5: *L. Entomophila* (Shandong); 6: *L. bostrychophila* (Chongqing); 7: *L. paeta* (Shandong); 8: *L. corrodens* (USA); 9: *L. Brunnea* (Prague); 10: *L. Rufa* (USA); 11: *L. mendax* (Jiangsu); 12: *L. Pearmani* (USA); 13: *L. Tricolor* (Shandong); CK: Negative control)

Figure S4 The electrophoresis strip for specificity estimation of species-specific primers (LPa15F and LPa180R) of *L. paeta* (M: DNA Marker Ⅱ; 1: *L. paeta* (Caoxian, Shandong); 2: *L. paeta* (Taian, Shandong); 3: *L. paeta* (Zhejiang); 4: *L. paeta* (Hubei); 5: *L. paeta* (USA); 6: *L. entomophila* (Shandong); 7: *L. bostrychophila* (Guangxi); 8: *L. decolor* (Yunnan); 9: *L. corrodens* (Prague); 10: *L. brunnea* (USA); 11: *L. Rufa* (USA); 12: *L. pearmani* (USA); 13: *L. mendax* (Jiangsu); 14: *L. tricolor* (Shandong); CK: Negative control)

Figure S5 The electrophoresis strip for specificity estimation of species-specific primers (LC170F and LC277R) of *L. corrodens* (M: D2000 DNA Marker; 1: *L. corrodens* (Danmark); 2: *L. corrodens* (Prague); 3: *L. corrodens* (USA); 4: *L. entomophila* (Shandong); 5: *L. bostrychophila* (Guangzhou); 6: *L. decolor* (Chongqing); 7: *L. paeta* (Hubei); 8: *L. brunnea* (USA); 9: *L. rufa* (USA); 10: *L. tricolor* (Shandong); 11: 11: *L. mendax* (Jiangsu); 12: *L. pearmani* (USA); CK: Negative control)

Figure S6 The electrophoresis strip for specificity estimation of species-specific primers (LBr350F and LBr577R) of *L. brunnea* (M: D2000 DNA Marker; 1: *L. brunnea* (Prague); 2: *L. brunnea* (USA); 3: *L. entomophila* (Beijiing); 4: *L.*

bostrychophila (Guangzhou); 5: *L. decolor* (USA); 6: *L. paeta* (Shandong); 7: *L. corrodens* (USA); 8: *L. rufa* (USA); 9: *L. pearmani* (USA); 10: *L. tricolor* (Shandong); 11: *L. mendax* (Jiangsu); CK: Negative control)

Figure S7 The electrophoresis strip for specificity estimation of species-specific primers (78LRuF-3 and 276LRuR-3) of *L. rufa* (M: D2000 DNA Marker Ⅱ; 1, 2, and 3: *L. rufa* (USA); 4: *L. entomophila* (Beijiing); 5: *L. bostrychophila* (Guangxi); 6: *L. decolor* (Yunnan); 7: *L. paeta* (Prague); 8: *L. corrodens* (USA); 9: *L. brunnea* (USA); 10: *L. mendax* (Jiangsu); 11: *L. pearmani* (USA); 12: *L. tricolor* (Shandong); CK: Negative control)

Figure S8 The electrophoresis strip for specificity estimation of species-specific primers (186LPeF and 436LPeR) of *L. pearmani* (M: D2000 DNA Marker; 1 and 2: *L. pearmani*(USA); 3: *L. entomophila* (Beijiing); 4: *L. bostrychophila* (Guangxi); 5: *L. decolor* (Yunnan); 6: *L. paeta* (Prague); 7: *L. corrodens* (USA); 8: *L. brunnea* (USA); 9: *L. rufa* (USA); 10: *L. mendax* (Jiangsu); 11: *L. tricolor* (Shandong); CK: Negative control)

Figure S9 The electrophoresis strip for specificity estimation of species-specific primers (LM60F and LM224R) of *L. mendax* (M: D2000 DNA Marker; 1 and 2: *L. mendax* (Jiangsu); 3: *L. entomophila* (Beijiing); 4: *L. bostrychophila* (Guangxi); 5: *L. decolor* (Yunnan); 6: *L. paeta* (Prague); 7: *L. corrodens* (USA); 8: *L. brunnea* (USA); 9: *L. rufa* (USA); 10: *L. pearmani* (USA); 11: *L. tricolor* (Shandong); CK: Negative control)

Figure S10 The electrophoresis strip for specificity estimation of species-specific primers (LTri20F and LTri249R) of *L. tricolor* (M: D2000 DNA Marker; 1 and 2: *L. tricolor* (Shandong); 3: *L. entomophila* (Beijiing); 4: *L. bostrychophila* (Guangxi); 5: *L. decolor* (Yunnan); 6: *L. paeta* (Prague); 7: *L. corrodens* (USA); 8: *L. brunnea* (USA); 9: *L. rufa* (USA); 10: *L. mendax* (Jiangsu); 11: *L. pearmani* (USA); CK: Negative control)

Figure S11 The sensitivity estimation of species-specific primers with different concentration of template DNA of *L. bostrychophila* (M: DNA Marker Ⅱ; 1: 40ng; 2: 20ng; 3: 10ng; 4: 1ng; 5: 0.1ng; 6: 0.01ng; 7:0.001 ng; 8: Negative control)

Figure S12 The sensitivity estimation of species-specific primers with different

concentration of template DNA of *L. entomophila* (M: D2000 DNA Marker; 1: 40ng; 2: 20ng; 3: 10ng; 4: 1ng; 5: 0.1ng; 6: 0.01ng; 7:0.001 ng; 8: Negative control)

Figure S13 The sensitivity estimation of species-specific primers with different concentration of template DNA of *L. decolor* (M: D2000 DNA Marker; 1: 40ng; 2: 20ng; 3: 10ng; 4: 1ng; 5: 0.1ng; 6: 0.01ng; 7:0.001 ng; 8: Negative control)

Figure S14 The sensitivity estimation of species-specific primers with different concentration of template DNA of *L. paeta* (M: D2000 DNA Marker; 1: 40ng; 2: 20ng; 3: 10ng; 4: 1ng; 5: 0.1ng; 6: 0.01ng; 7:0.001 ng; 8: Negative control)

Figure S15 The sensitivity estimation of species-specific primers with different concentration of template DNA of *L. corrodens* (M: D2000 DNA Marker; 1: 40ng; 2: 20ng; 3: 10ng; 4: 1ng; 5: 0.1ng; 6: 0.01ng; 7:0.001 ng; 8: Negative control)

Figure S16 The sensitivity estimation of species-specific primers with different concentration of template DNA of *L. brunnea* (M: D2000 DNA Marker; 1: 40ng; 2: 20ng; 3: 10ng; 4: 1ng; 5: 0.1ng; 6: 0.01ng; 7:0.001 ng; 8: Negative control)

Figure S17 The sensitivity estimation of species-specific primers with different concentration of template DNA of *L. rufa* (M: D2000 DNA Marker; 1: 40ng; 2: 20ng; 3: 10ng; 4: 1ng; 5: 0.1ng; 6: 0.01ng; 7:0.001 ng; 8: Negative control)

Figure S18 The sensitivity estimation of species-specific primers with different concentration of template DNA of *L. pearmani* (M: D2000 DNA Marker; 1: 40ng; 2: 20ng; 3: 10ng; 4: 1ng; 5: 0.1ng; 6: 0.01ng; 7:0.001 ng; 8: Negative control)

Figure S19 The sensitivity estimation of species-specific primers with different concentration of template DNA of *L. mendax* (M: D2000 DNA Marker; 1: 40ng; 2:

20ng; 3: 10ng; 4: 1ng; 5: 0.1ng; 6: 0.01ng; 7:0.001 ng; 8: Negative control)

Figure S20 The sensitivity estimation of species-specific primers with different concentration of template DNA of *L. tricolor* (M: D2000 DNA Marker; 1: 40ng; 2: 20ng; 3: 10ng; 4: 1ng; 5: 0.1ng; 6: 0.01ng; 7:0.001 ng; 8: Negative control)

Table S1 The primers for the ITS2 gene amplification of 10 stored-product psocids

Primer	名称	sequence
ITS2 rDNA	Upstream primer	5’- TGTGAACTGCAGGACACATG-3’
ITS2 rDNA	Downstream primer	5’- GTCTTGCCTGCTCTGAG-3’
ITS2 rDNA	Downstream primer	5’-GTCTTGTCTGATCTGAG-3’

Table S2 The ITS2 gene sequence of 10 stored-product psocids

(1) *L. entomophila*

AACATCGATATTTCGAACGC**ACATTGCTGGGTTAGGATTCA**TTCCGGCCCAACGTCTGTCTGAGGGTCGGTGATCAATATTAAACCGAACGTTCCGTGTGATACGGAACTGATGGGAAGCTCGAAGTAAGTCGATTTCCGAAACGTAGAAAAATGACGGGTAGAGGAAAGAAAAAGCGAGTGCACGC**ACAAATTACAGAGTTCGGAAG**AACTGTGCTTGCAGTGGTTACATTTTTAACGTCACGTTAGTAAGCGTAAAACTTTACCACGGACGTATAAAAGAACAACGAATTCTATATCTACTCTTCATCGTC

(2) *L. bostrychophila*

AACATCGATATTTCGAA**CGCACATTGCCGAGCTAGGAT**TTTTTCCGGCTCGACGTCTGTCTGAGGGTCGATAATTTTTATTAAAATAAAGTTTTCGTTGAGAATAAAATCTCACGGACTGTGGAAGTGTCGAAAGATTTGAGTTTTGCTCGAAACTTCGTCTTCTGAAAGTTA**AGAACGAGAATACAAGGAGCG**AGCTCGTATTTTGCGGTTTTCTGCTTCACGTTAGTTTTGTTTTACTGAGCGGATTGTCAGAAAAATCGTAAGAGTTCCTTTTGTAATCTTTTCATCGTC

(3) *L. decolor*

AACATCGATATTTCGAACGCACATTGCGCTCTGGGGAACTCCGTTCCCGGAGCACGTCTGTCTGAGGGTCGGTGCTTAAATATAAATTTCGGAACGATTCTTTTTAACGGAAGGTCGTGATCGAAACGTTGAAAGCTTCGGGTCGTAAGAAAACCCGTCTTTC**GAAATGCACTAGAACCGAGA**ATCTTCGTAAATGAAGAAGGCGAGAGCTGTAAAGAGCAGCGATACGTGAATCACGTTTACCGACAGGCGAACGATAACAAGCATGCCCTAAGCACGTCTTGTAAAAAAAGATTCGAATGCATCGCATCGATCTTT**ACTCGTCACGTTCAATATAT**ATATAAGTAACGGGCGAGAGTGCTTTAGGGGAAGCGGTTCGCATATATTTTTATAACTGTCGTATACGCGGAAACGATGATCGCTAAGCTCGTTTAAAATATTTTTTTTTGCCCTCGTCTTTTTTTTCGAAACGAAAAAGATTCTTTTCATCGTC

(4) *L. paeta*

AACATCGATATTTC**GAACGCACATTGCCGAGTTTT**GGATTTATCCTGACTCGACGTCTGTCTGAGGGTCGGTGATATCTATTGAAAGATATTTCGTTGTTTACCGACGATTTTGAGAGTGTCAGATTCTGCATTGCAGAATTTGTCTTTTGAAATACGATGTCACGAGGTTCTTGCAA**AAGGGTAACTGCATCGGACGGT**ATTTGGTGTCCAATGCTTTCGACTTTCGGAGGTCACGTTAGTATGATTTTACGAAACGGATTTGGAAAGTCGAGATATTTGCAAAATCTCATCATCGTC

(5) *L. corrodens*

AACATCGATATTTCGAACGCACATTGCCGAGCTAGGATTTTTTCCGGCTCGACGTCTGTCTGAGGGTCGATGATTTCTAATAAACAGATTTTCCGTTAGAGTCAATCTATGGAAAAATT

GAAAGCCTCGAATTATGTATAATAATTCGTCTTTCGAAATAAAATGTACC**CGAGGAAAT**
CTCACAAGACGAAAGCAAATTTGTGGCAAAATGAATATAGGACGATATATATCGTCTTT
GCTGCAAAAACGGTTTCTCTGCGTCACGTTAGTATTAAT**ACCAAACGGATTGTAGAGA**
ACTGTATTATTGTGAGGATTCTCTTCATCGTC

(6) *L. brunnea*
AACATCGATATTTCGAACGCACATTGCGCTCTGGGGAACTCCGTTCCCGGAGCACGTCT
GTCTGAGGGTCGATGCTTACTTGTAAAATTTAGAACGCTGGTTATTTTTTTTCGAATCGC
AAACGATTGAAAAAAAAATCTTTTTAACCGGCGTGATAAAATGATGAAAGCTTCGGGT
CGCTCTCCGCGTAAGCGGAGGGTTCCGTCTTTCGAAATGCACGATCCCGAGATATCGCT
CGGAAATAAAACGAGTTCGAAGAAGACCGATACGTATAATAGTCTCGAGCCGCCAGCG
GCGAAAGCGATCACGTCTTGTTTTTTTTACGTGCCCGCGATACGATGCGTTTTGA**ACCG**
AGATCCTTGTTACGAATGCACAAACGTAATAATAATAATAAGGATCGAAGAAGGTCTT
CCTCTCGTCACGTTAGTGGTTATTTTGATATGTAACGTGAAACGGTCGAGATGGAAAAC
GTATCGCGGGTAAAGACGAGAAAAAGAGGAGGCTTTCGAACCGTGTTTTTATCGCGGC
TCTCGGAAACGAAGATCGGTTTTCGTGTACAAGCGTTTGTTAGTAGTA**CTCGTAAAAC**
AGAATTCCGGCGATTTCTTTTCATCGTC

(7) *L. rufa*
AACATCGATATTTCGAACGCACATTGCGCTCTGGGGAACTCCGTTCCCGGAGCACGTCT
GTCTGAGGGTCGGTGCTT**AATGTTAAATGTCGGAACGCT**TGACGGCGTGATCGAAAC
GTTGAAAGCTTCGGGTCGTGAAAATTAAGACCCGTCTTTCCAAATGCACGAAAGACCG
AGAATCTTATGAGGAAGAAGAACACTATAGAGAACGATACGTCAATCTCACGGCTGCT
CGTAAAGCAAATTAAAAGTACAT**TAGCGTACCGATAGGTCGTAA**ATTTTTTTTTAAAG
CGAGCCCGTAAGTAAACGAAAAAGAAAAGATCGCACGCATCGTCGTCGATCTTCCTCG
TCACGTTCATGATGATACATAGCAAATAAATAAATAAAAGAAAAACGGTCGAGGGTACT
GCGGGGATTCGGTATAGCGTGAGGAAACGAAAATCGTTACTCGTCGAAATTGTGTTTTT
TATTTTTTTTTCACCTCAAGAAAAGATTCTTTTCATCGTC

(8) *L. pearmani*
AACATCGATATTTCGAACGCACATTGCGCTCTGGGGAACTCCGTTCCCGGAGCACGTCT
GTCTGAGGGTCGATGCTTAATTGTAAAATCTAGAACGCCGTCTCTCGCACGATTAAGTT
CGGGGGGAGCGGTCGTGATAAAAGACTCCGTTGAATGCTTCGAGCTCGCGAGAGCCC
GTCATTCCAA**ATGCACGAAACGAGATTCTCA**CAAGAATTTCGAACGAGTCGGAAGAT
GAGAGAAACGATACGTAAATCGTACTTGCTATCTACGGAAGCCAATATTTGCCCGTGAT
ACGAAACATGTCTTCCTCGTCACGTTAGTCAGTTTGTTAATAATAAGCAAAATAACGGG
CGAGACGTTGATGTATCGCCGCGGCGGCTAGCAGTAGATCGTACGGAAACGAAAATCG
TTTACTCG**TTTGAAGAATGGTAGCCGTT**TTTCGTTGGTAGCCTTTCGACCAAACCGAA
GTGAGATTCTTTTCATCGTC

(9) *L. mendax*

AACATCGATATTTCGAACGCACATTGCGGTCCTTGGAACTTTGTTCCCGGACCACGTCT**GTCTGAGGGTCGGTGTTTGCT**ATTAGAACATTTTTTATTCGTTTCGCTTCGGCACGAATAATGTGGAGGTTTCGAGTATCGGCTCGTCTTCCTAAACAGAATAATGCGAGAATTCCGTAAGCGGGAATTTATGGGTTTTGTCCTCGCGTCACGTTTCTGAAATT**AAGAGCAGCGTTGACGGACTT**GCGAGGTAAAGCTCTGTTCGGCGAAATAGACGGAAATTCTAATTCATCGTC

(10) *L. tricolor*

AACATCGATATTTCGAACG**CACATTGCCGAACTTTGAATT**TATTCTGGTTCGACGTCTGTCTGAGGGTCGATGAAAATTATTGAAATATTTACCGTTAGATGTAATGTCTACGGTTATTGAAAGTTACGAATTTCCTCGGAAATTCGTCTTTCGAAATTCATATGTCCCCGAGAATCGTACATGAAAGAGAATGTCTCAGTAAATGGTATGTTATTTGCTGCATTGGGCATATTTCTGCTTCACGTTA**GTAAACCAAACGGATAGTAGA**AAAATGTCTTCGTGGAACGATTTCTCATCTTCGTCCTCAGATCAGGAATCACTAGTGAATTCGCGGCCGCCTGCAGGTC

Table S3 The number of 53 specific sequences submitted to Genebank

Population	Locations	Authors	No. GenBank
*L. entomophila*_BJ- P. R. China	Beijing,P. R.China	Cui Bingyi, Li Zhihong	KC707588
*L. entomophila*_HuB- P. R. China	HuBei,P. R. China	Cui Bingyi, Li Zhihong	KC707590
*L. entomophila*_GX- P. R. China	Guangxi,P. R.China	Cui Bingyi, Li Zhihong	KC707589
*L. entomophila*_SD- P. R. China	Shandong,P. R.China	Cui Bingyi, Li Zhihong	KC707591
*L. bostrychophila*_BJ- P. R. China	Beijing,P. R.China	Cui Bingyi, Li Zhihong	JQ966091
*L. bostrychophila*_GX- P. R. China	Guangxi,P. R.China	Cui Bingyi, Li Zhihong	JQ966093
*L. bostrychophila*_GZ- P. R. China	Guangzhou,P.R.China	Cui Bingyi, Li Zhihong	JQ966096
*L.bostrychophila*_HeN- P. R. China	Henan,P. R.China	Cui Bingyi, Li Zhihong	JQ966097
*L. bostrychophila*_CQ- P. R. China	Chongqing,P. R.China	Cui Bingyi, Li Zhihong	JQ966099
*L. bostrychophila*_P-CZ	Prague,CZ	Cui Bingyi, Li Zhihong	JQ966092
*L. bostrychophila*_CZ	Central Bohemia, CZ	Cui Bingyi, Li Zhihong	JQ966095
*L. bostrychophila*_USA	Manhattan,USA	Cui Bingyi, Li Zhihong	JQ966094
*L. bostrychophila*_GER	Berlin ,GER	Cui Bingyi, Li Zhihong	JQ966098
*L. decolor*_CQ- P. R. China	Chongqing,P. R.China	Cui Bingyi, Li Zhihong	KC707592
*L. decolor*_YN- P. R. China	Yunnan, P. R.China	Cui Bingyi, Li Zhihong	KC707593
*L. decolor*_P-CZ	Prague,CZ	Cui Bingyi, Li Zhihong	KC707594
*L. decolor*_P-CZ	Prague,CZ	Cui Bingyi, Li Zhihong	KC707595
*L. decolor*_P-CZ	Prague,CZ	Cui Bingyi, Li Zhihong	KC707596
*L. decolor*_USA	USA	Cui Bingyi, Li Zhihong	KC707597
*L. decolor*_USA	USA	Cui Bingyi, Li Zhihong	KC707598
*L. decolor*_USA	USA	Cui Bingyi, Li Zhihong	KC707599
*L. paeta*_USA	USA	Cui Bingyi, Li Zhihong	GU563533
*L. paeta*_HeB-P. R.China	HebeiP,R.China	Cui Bingyi, Li Zhihong	KC707604
*L. paeta*_SDT-P. R.China	Taian,Shandong,P.R.China	Cui Bingyi, Li Zhihong	KC707605
*L. paeta*_SDT-P. R.China	Taian,Shandong,P.R.China	Cui Bingyi, Li Zhihong	KC707606
*L. paeta*_SDT-P. R.China	Taian,Shandong,P.R.China	Cui Bingyi, Li Zhihong	KC707607
*L. paeta*_SDC-P. R.China	Caoxian,Shandong,P.R.China	Cui Bingyi, Li Zhihong	KC707619
*L. paeta*_ZJ-P. R.China	Zhejiang,P. R.China	Cui Bingyi, Li Zhihong	KC707612
*L. paeta*_ZJ-P. R.China	Zhejiang,P. R.China	Cui Bingyi, Li Zhihong	KC707613
*L. paeta*_HuB-P. R.China	Hubei,P. R.China	Cui Bingyi, Li Zhihong	KC707614
*L. paeta*_HuB-P. R.China	Hubei,P. R.China	Cui Bingyi, Li Zhihong	KC707615
*L. paeta*_HeN-P. R.China	Henan,P. R.China	Cui Bingyi, Li Zhihong	KC707616
*L. paeta*_HeN-P. R.China	Henan,P. R.China	Cui Bingyi, Li Zhihong	KC707617
*L. paeta*_HeN-P. R.China	Henan,P. R.China	Cui Bingyi, Li Zhihong	KC707618
*L. paeta*_CZ	Prague,CZ	Cui Bingyi, Li Zhihong	KC707603

*L. paeta*_USA	USA	Cui Bingyi, Li Zhihong	KC707608
*L. paeta*_USA	USA	Cui Bingyi, Li Zhihong	KC707609
*L. paeta*_USA	USA	Cui Bingyi, Li Zhihong	KC707610
*L. paeta*_USA	USA	Cui Bingyi, Li Zhihong	KC707611
*L. corrodens*_CZ	Prague,CZ	Cui Bingyi, Li Zhihong	KC707623
*L. corrodens*_DMK.	Danmark	Cui Bingyi, Li Zhihong	KC707620
*L. corrodens*_DMK.	Danmark	Cui Bingyi, Li Zhihong	KC707621
*L. corrodens*_DMK.	Danmark	Cui Bingyi, Li Zhihong	KC707622
*L. corrodens*_ USA	USA	Cui Bingyi, Li Zhihong	KC707624
*L. corrodens*_USA	USA	Cui Bingyi, Li Zhihong	KC707625
*L. brunnea*_P-CZ	Prague,CZ	Cui Bingyi, Li Zhihong	KC707626
*L. brunnea*_USA	USA	Cui Bingyi, Li Zhihong	KC707626
*L. mendax*_JS-P. R. China	Jiangsu,P. R.China	Cui Bingyi, Li Zhihong	KC707629
*L. pearmani*_USA	USA	Cui Bingyi, Li Zhihong	KC707628
*L. tricolor*_SD-P. R. China	Shandong,P. R.China	Cui Bingyi, Li Zhihong	KC707630
*L. tricolor*_SD-P. R. China	Shandong,P. R.China	Cui Bingyi, Li Zhihong	KC707631
*L. tricolor*_SD-P. R. China	Shandong,P. R.China	Cui Bingyi, Li Zhihong	KC707632
*L. rufa*_USA	USA	Cui Bingyi, Li Zhihong	KC707629

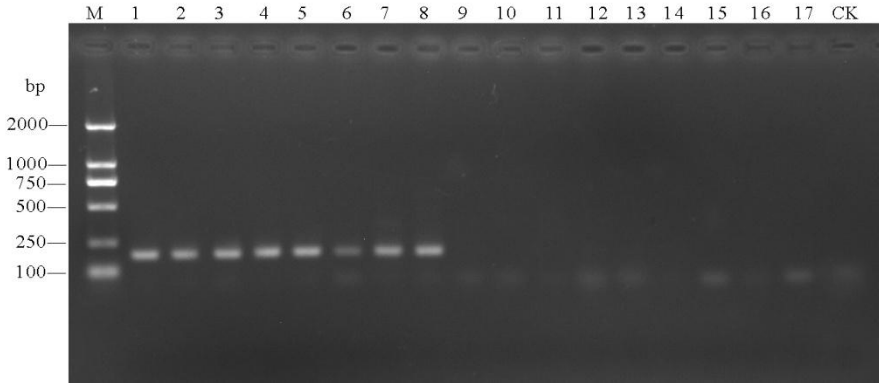

Figure S1 The electrophoresis strip for specificity estimation of species-specific primers (LBF and LBR) of *L. bostrychophila* (M: D2000 DNA Marker; 1: *L. bostrychophila* (Guangxi); 2: *L. bostrychophila* (Henan); 3: *L. bostrychophila* (Chongqing); 4: *L. bostrychophila* (Berlin); 5: *L. bostrychophila* (USA); 6: *L. bostrychophila* (Prague); 7 *L. bostrychophila* (Beijing); 8: *L. bostrychophila* (Guangzhou); 9: *L. Entomophila* (Shandong); 10: *L. Decolor* (Yunnan); 11: *L. paeta* (Zhejiang); 12: *L. corrodens* (USA); 13: *L. Brunnea* (Prague); 14: *L. Rufa* (USA); 15: *L. Pearmani* (USA); 16: *L. mendax* (Jiangsu); 17: *L. Tricolor* (Shandong); CK: Negative control)

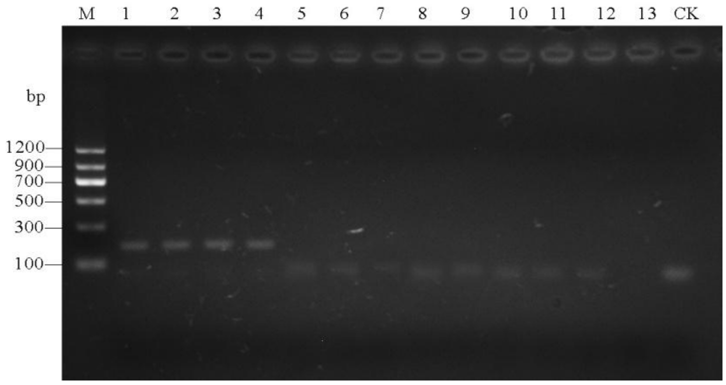

Figure S2 The electrophoresis strip for specificity estimation of species-specific primers (21LEnF and 208LEnR-2) of *L. Entomophila* (M: DNA Marker Ⅱ; 1: *L. entomophila* (Beijing); 2: *L. entomophila* (Guangxi); 3: *L. entomophila* (Prague); 4: *L. entomophila* (Chongqing); 5: *L. bostrychophila* (Guangxi); 6: *L. bostrychophila* (Yunnan); 7: *L. Decolor* (Yunnan); 8: *L. corrodens* (USA); 9: *L. brunnea* (Prague); 10: *L. rufa* (USA); 11: *L. pearmani* (USA); 12: *L. mendax* (Jiangsu); 13: *L. tricolor*(Shandong); CK: Negative control)

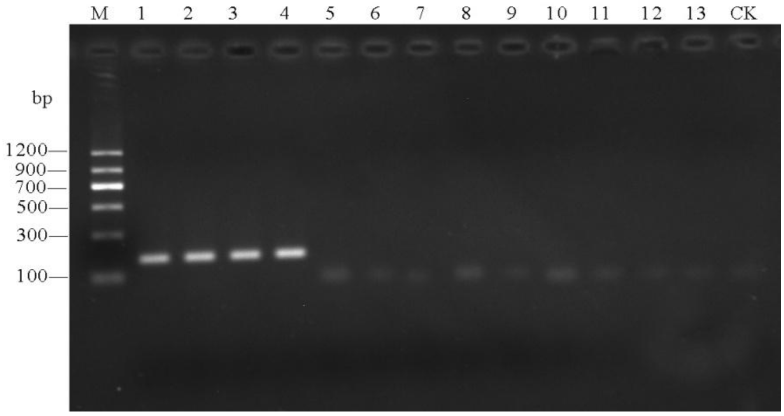

Figure S3 The electrophoresis strip for specificity estimation of species-specific primers (164LDeF and 319LDeR) of *L. decolor* (M: DNA Marker Ⅱ; 1 and 2: *L. Decolor* (Yunnan); 3 and 4: *L. Decolor* (Chongqing); 5: *L. Entomophila* (Shandong); 6: *L. bostrychophila* (Chongqing); 7: *L. paeta* (Shandong); 8: *L. corrodens* (USA); 9: *L. Brunnea* (Prague); 10: *L. Rufa* (USA); 11: *L. mendax* (Jiangsu); 12: *L. Pearmani* (USA); 13: *L. Tricolor* (Shandong); CK: Negative control)

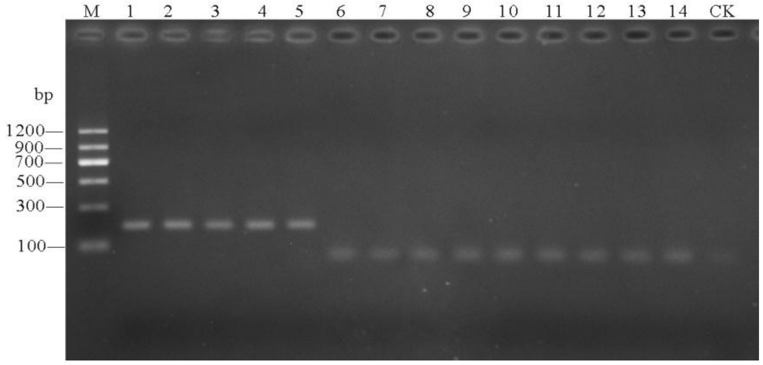

Figure S4 The electrophoresis strip for specificity estimation of species-specific primers (LPa15F and LPa180R) of *L. paeta* (M: DNA Marker Ⅱ; 1: *L. paeta* (Caoxian, Shandong); 2: *L. paeta* (Taian, Shandong); 3: *L. paeta*(Zhejiang); 4: *L. paeta* (Hubei); 5: *L. paeta* (USA); 6: *L. entomophila* (Shandong); 7: *L. bostrychophila* (Guangxi); 8: *L. decolor* (Yunnan); 9: *L. corrodens* (Prague); 10: *L. brunnea* (USA); 11: *L. Rufa* (USA); 12: *L. pearmani* (USA); 13: *L. mendax* (Jiangsu); 14: *L. tricolor* (Shandong); CK: Negative control)

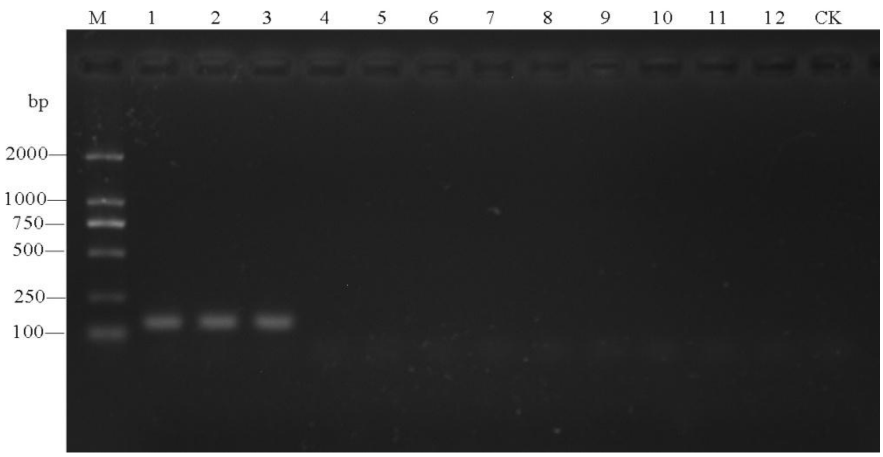

Figure S5 The electrophoresis strip for specificity estimation of species-specific primers (LC170F and LC277R) of *L. corrodens* (M: D2000 DNA Marker; 1: *L. corrodens* (Danmark); 2: *L. corrodens* (Prague); 3: *L. corrodens* (USA); 4: *L. entomophila* (Shandong); 5: *L. bostrychophila* (Guangzhou); 6: *L. decolor* (Chongqing); 7: *L. paeta* (Hubei); 8: *L. brunnea* (USA); 9: *L. rufa* (USA); 10: *L. tricolor* (Shandong); 11: 11: *L. mendax* (Jiangsu); 12: *L. pearmani* (USA); CK: Negative control)

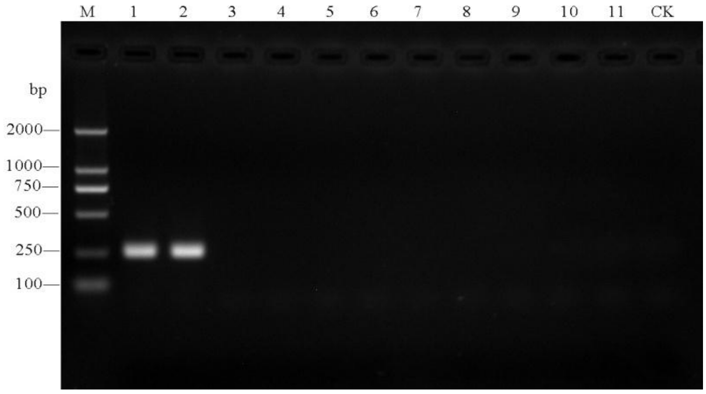

Figure S6 The electrophoresis strip for specificity estimation of species-specific primers (LBr350F and LBr577R) of *L. brunnea* (M: D2000 DNA Marker; 1: *L. brunnea* (Prague); 2: *L. brunnea* (USA); 3: *L. entomophila* (Beijiing); 4: *L. bostrychophila* (Guangzhou); 5: *L. decolor* (USA); 6: *L. paeta* (Shandong); 7: *L. corrodens* (USA); 8: *L. rufa* (USA); 9: *L. pearmani* (USA); 10: *L. tricolor* (Shandong); 11: *L. mendax* (Jiangsu); CK: Negative control)

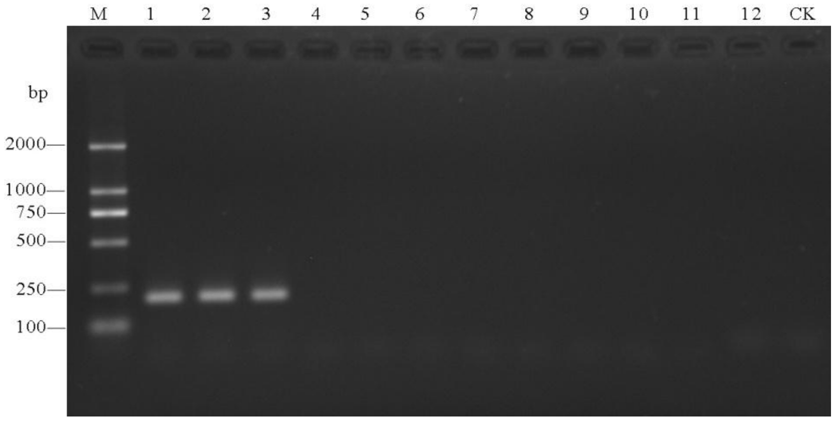

Figure S7 The electrophoresis strip for specificity estimation of species-specific primers (78LRuF-3 and 276LRuR-3) of *L. rufa* (M: D2000 DNA Marker Ⅱ; 1, 2, and 3: *L. rufa* (USA); 4: *L. entomophila* (Beijiing); 5: *L. bostrychophila* (Guangxi); 6: *L. decolor* (Yunnan); 7: *L. paeta* (Prague); 8: *L. corrodens* (USA); 9: *L. brunnea* (USA); 10: *L. mendax* (Jiangsu); 11: *L. pearmani* (USA); 12: *L. tricolor* (Shandong); CK: Negative control)

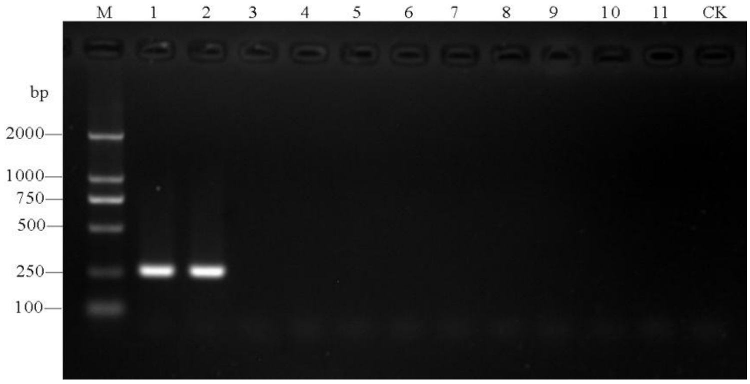

Figure S8 The electrophoresis strip for specificity estimation of species-specific primers (186LPeF and 436LPeR) of *L. pearmani* (M: D2000 DNA Marker; 1 and 2: *L. pearmani*(USA); 3: *L. entomophila* (Beijiing); 4: *L. bostrychophila* (Guangxi); 5: *L. decolor* (Yunnan); 6: *L. paeta* (Prague); 7: *L. corrodens* (USA); 8: *L. brunnea* (USA); 9: *L. rufa* (USA); 10: *L. mendax* (Jiangsu); 11: *L. tricolor* (Shandong); CK: Negative control)

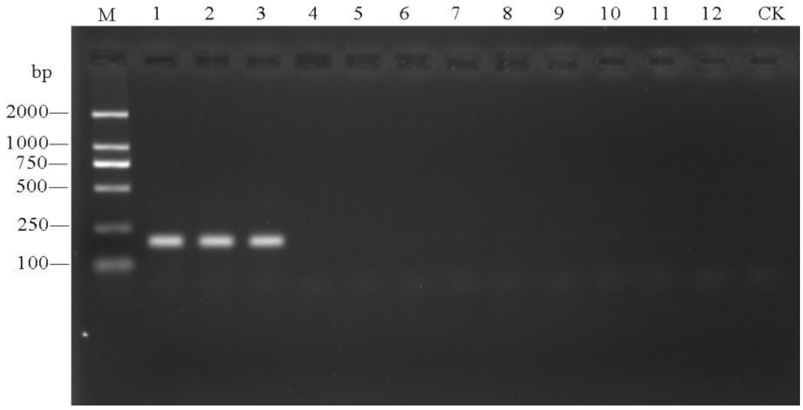

Figure S9 The electrophoresis strip for specificity estimation of species-specific primers (LM60F and LM224R) of *L. mendax* (M: D2000 DNA Marker; 1 and 2: *L. mendax* (Jiangsu); 3: *L. entomophila* (Beijiing); 4: *L. bostrychophila* (Guangxi); 5: *L. decolor* (Yunnan); 6: *L. paeta* (Prague); 7: *L. corrodens* (USA); 8: *L. brunnea* (USA); 9: *L. rufa* (USA); 10: *L. pearmani* (USA); 11: *L. tricolor* (Shandong); CK: Negative control)

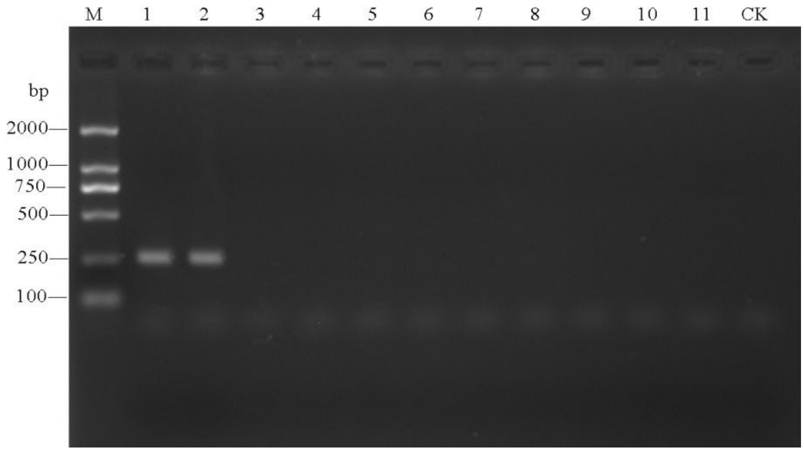

Figure S10 The electrophoresis strip for specificity estimation of species-specific primers (LTri20F and LTri249R) of *L. tricolor* (M: D2000 DNA Marker; 1 and 2: *L. tricolor* (Shandong); 3: *L. entomophila* (Beijiing); 4: *L. bostrychophila* (Guangxi); 5: *L. decolor* (Yunnan); 6: *L. paeta* (Prague); 7: *L. corrodens* (USA); 8: *L. brunnea* (USA); 9: *L. rufa* (USA); 10: *L. mendax* (Jiangsu); 11: *L. pearmani* (USA); CK: Negative control)

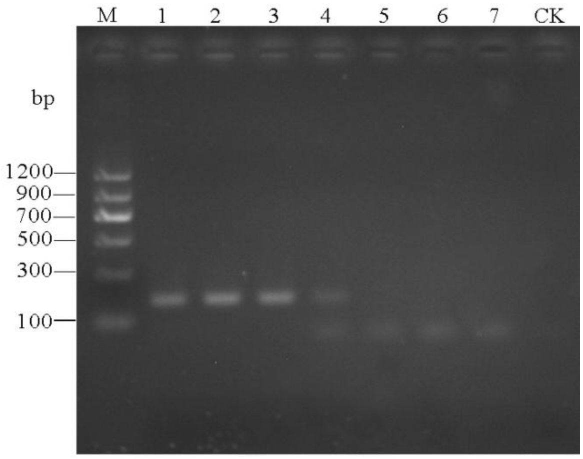

Figure S11 The sensitivity estimation of species-specific primers with different concentration of template DNA of *L. bostrychophila* (M: DNA Marker Ⅱ; 1: 40ng; 2: 20ng; 3: 10ng; 4: 1ng; 5: 0.1ng; 6: 0.01ng; 7:0.001 ng; 8: Negative control)

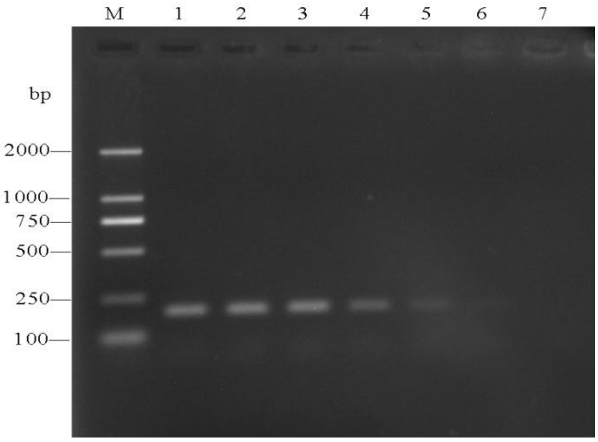

Figure S12 The sensitivity estimation of species-specific primers with different concentration of template DNA of *L. entomophila* (M: D2000 DNA Marker; 1: 40ng; 2: 20ng; 3: 10ng; 4: 1ng; 5: 0.1ng; 6: 0.01ng; 7:0.001 ng; 8: Negative control)

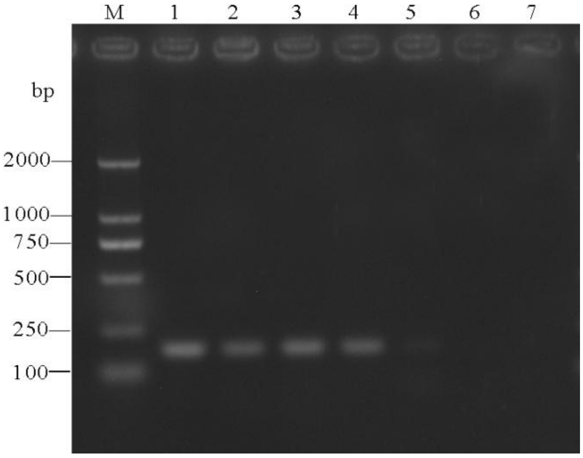

Figure S13 The sensitivity estimation of species-specific primers with different concentration of template DNA of *L. decolor* (M: D2000 DNA Marker; 1: 40ng; 2: 20ng; 3: 10ng; 4: 1ng; 5: 0.1ng; 6: 0.01ng; 7:0.001 ng; 8: Negative control)

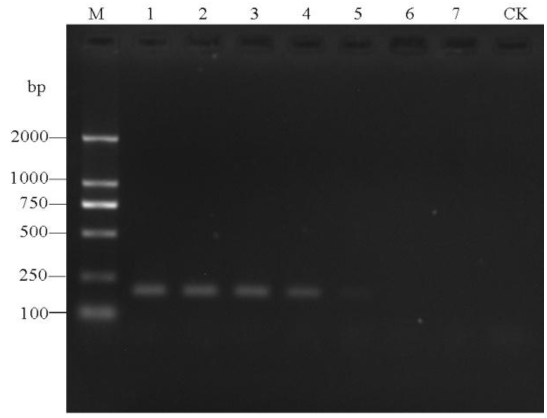

Figure S14 The sensitivity estimation of species-specific primers with different concentration of template DNA of *L. paeta* (M: D2000 DNA Marker; 1: 40ng; 2: 20ng; 3: 10ng; 4: 1ng; 5: 0.1ng; 6: 0.01ng; 7:0.001 ng; 8: Negative control)

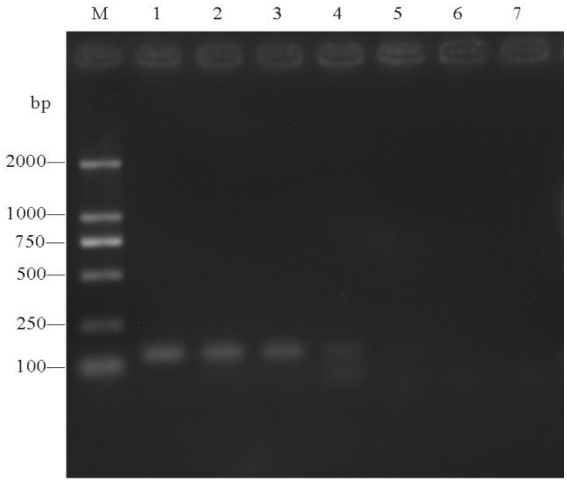

Figure S15 The sensitivity estimation of species-specific primers with different concentration of template DNA of *L. corrodens* (M: D2000 DNA Marker; 1: 40ng; 2: 20ng; 3: 10ng; 4: 1ng; 5: 0.1ng; 6: 0.01ng; 7:0.001 ng; 8: Negative control)

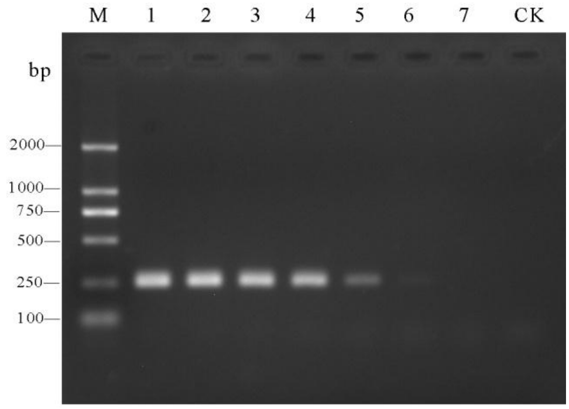

Figure S16 The sensitivity estimation of species-specific primers with different concentration of template DNA of *L. brunnea* (M: D2000 DNA Marker; 1: 40ng; 2: 20ng; 3: 10ng; 4: 1ng; 5: 0.1ng; 6: 0.01ng; 7:0.001 ng; 8: Negative control)

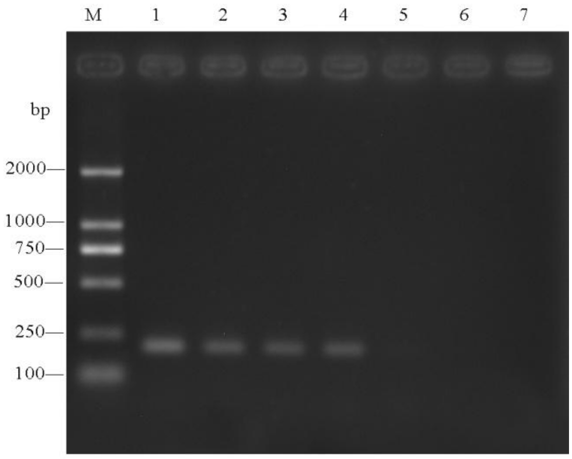

Figure S17 The sensitivity estimation of species-specific primers with different concentration of template DNA of *L. rufa* (M: D2000 DNA Marker; 1: 40ng; 2: 20ng; 3: 10ng; 4: 1ng; 5: 0.1ng; 6: 0.01ng; 7:0.001 ng; 8: Negative control)

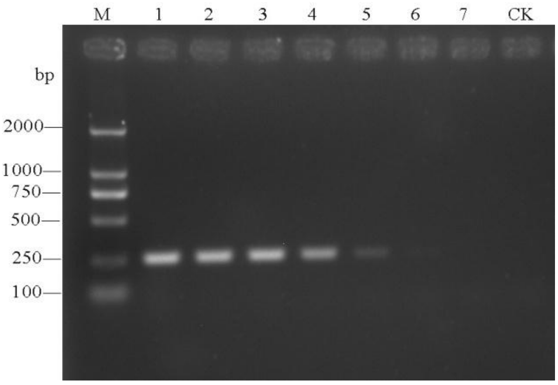

Figure S18 The sensitivity estimation of species-specific primers with different concentration of template DNA of *L. pearmani* (M: D2000 DNA Marker; 1: 40ng; 2: 20ng; 3: 10ng; 4: 1ng; 5: 0.1ng; 6: 0.01ng; 7:0.001 ng;

8: Negative control)

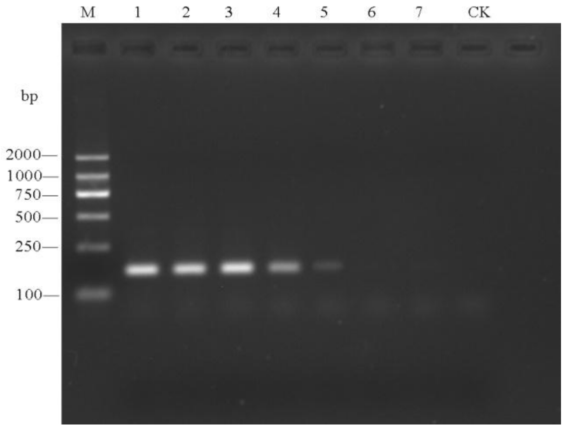

Figure S19 The sensitivity estimation of species-specific primers with different concentration of template DNA of *L. mendax* (M: D2000 DNA Marker; 1: 40ng; 2: 20ng; 3: 10ng; 4: 1ng; 5: 0.1ng; 6: 0.01ng; 7:0.001 ng; 8: Negative control)

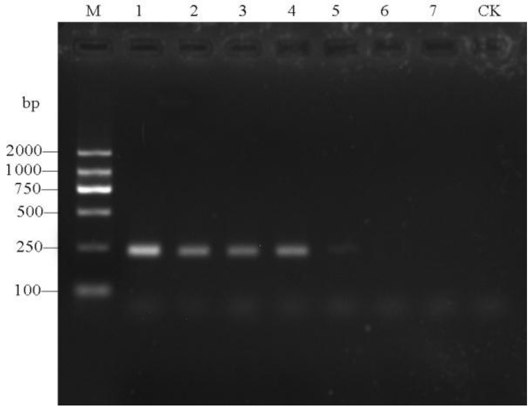

Figure S20 The sensitivity estimation of species-specific primers with different concentration of template DNA of *L. tricolor* (M: D2000 DNA Marker; 1: 40ng; 2: 20ng; 3: 10ng; 4: 1ng; 5: 0.1ng; 6: 0.01ng; 7:0.001 ng; 8: Negative control)
